# *Cdh11* Acts as a Tumor Suppressor in a Murine Retinoblastoma Model by Facilitating Tumor Cell Death

**DOI:** 10.1371/journal.pgen.1000923

**Published:** 2010-04-22

**Authors:** Mellone N. Marchong, Christine Yurkowski, Clement Ma, Clarellen Spencer, Sanja Pajovic, Brenda L. Gallie

**Affiliations:** 1 Campbell Family Institute for Cancer Research, Ontario Cancer Institute/Princess Margaret Hospital, University Health Network, Toronto, Ontario, Canada; 2 Department of Medical Biophysics, University of Toronto, Toronto, Ontario, Canada; 3 Department of Molecular Genetics, University of Toronto, Toronto, Ontario, Canada; 4 Department of Biostatistics, Ontario Cancer Institute/Princess Margaret Hospital, University Health Network, Toronto, Ontario, Canada; 5 Department of Ophthalmology, University of Toronto, Toronto, Ontario, Canada; Stanford Medical School, United States of America

## Abstract

*CDH11* gene copy number and expression are frequently lost in human retinoblastomas and in retinoblastomas arising in TAg-RB mice. To determine the effect of *Cdh11* loss in tumorigenesis, we crossed *Cdh11* null mice with TAg-RB mice. Loss of *Cdh11* had no gross morphological effect on the developing retina of *Cdh11* knockout mice, but led to larger retinal volumes in mice crossed with TAg-RB mice (p = 0.01). Mice null for *Cdh11* presented with fewer TAg-positive cells at postnatal day 8 (PND8) (p = 0.01) and had fewer multifocal tumors at PND28 (p = 0.016), compared to mice with normal *Cdh11* alleles. However, tumor growth was faster in *Cdh11*-null mice between PND8 and PND84 (p = 0.003). In tumors of *Cdh11-*null mice, cell death was decreased 5- to 10-fold (p<0.03 for all markers), while proliferation *in vivo* remained unaffected (p = 0.121). Activated caspase-3 was significantly decreased and β-catenin expression increased in *Cdh11* knockdown experiments *in vitro*. These data suggest that *Cdh11* displays tumor suppressor properties *in vivo* and *in vitro* in murine retinoblastoma through promotion of cell death.

## Author Summary

Despite over two decades since loss of *RB1* was implicated in initiating retinoblastoma, the unique tissue specificity of this process remains puzzling. Indeed, functional loss of both alleles of the *RB1* tumor suppressor gene results in >40,000-fold increase in predisposition to retinal cancer during childhood, while one constitutional *RB1* mutant allele confers a broader but much lower cancer predisposition later in life. We have proposed a specific signature of progressive genomic changes that leads to full tumor development. One of these changes is genomic loss of the *CDH11* gene, suggesting that this gene normally suppresses the development of retinoblastoma. We present novel data indicating that *Cdh11* functions as a tumor suppressor gene in retinoblastoma by facilitating cell death. Our insight into the sequence of events that contribute to retinoblastoma development is important for future therapies and fundamental understanding of cancer.

## Introduction

Retinoblastoma is initiated by loss of both *RB1* alleles, denoted M1 and M2 mutational events [1]. These initiating events are sufficient for the development of the benign tumor, retinoma, but not enough to drive to malignancy; additional mutational events (M3-Mn) are required for development to retinoblastoma [1]–[3]. Early cytogenetic analysis performed on human retinoblastoma samples revealed recurrent chromosomal abnormalities [4]. Five comparative genomic hybridization (CGH) studies and one matrix CGH study confirmed these results and identified common genomic regions of gain and loss in retinoblastoma tumors [5]–[10]. Based on the location of these genomic changes, potential oncogenes (*KIF14*, *E2F3* and *DEK*) and tumor suppressor genes (*p75^NTR^* and *CDH11*) have been identified and shown to have involvement in retinoblastoma development and progression [2], [11]–[13]. Based on the frequency of and correlation between these mutational events, we proposed a genetic cascade to malignancy. Subsequent to loss of *RB1*, the most frequent event is gain of 1q (involving *KIF14*) [11], followed by gain of 6p (involving *E2F3* and *DEK*) [12], [14], and then, loss of 16q (involving *CDH11*) [13] or gain of *MYCN* [15].

Understanding the pathway to tumorigenesis is important for the development of new and better therapeutics that can ultimately be used to halt retinoblastoma progression at an early stage. Importantly, delineating the order of mutational events in retinoblastoma, the prototypical model of cancer, is pertinent to the understanding of oncogenesis in general.

In previous work, we narrowed the minimal region of genomic loss on chromosomal arm 16q22.1 to *CDH11* [13]. This gene was lost in 58% of 71 retinoblastoma tumors, and its expression showed gradual loss in tumors of the murine retinoblastoma model (TAg-RB) induced by simian virus 40 large T-Antigen (TAg) expression [16], with some advanced tumors (3 of 8) showing loss of *Cdh11*. Thus, we proposed that *Cdh11* acts as a tumor suppressor gene in retinoblastoma.

Gratias et al, 2007, identified a complex pattern of 16q loss of heterozygosity (LOH) in 18 out of 58 retinoblastoma samples. One tumor showed LOH at 16q24, the region where *CDH13* is located; however, *CDH13* did not show reduced expression in retinoblastoma tumors, confirming our previous findings [13], [17]. Gratias et al, 2007 did not test markers for *CDH11* directly, as it was outside their minimal region of loss. They also correlated 16q allelic loss with diffuse intraocular seeding, implicating 16q loss as a late mutational event, in agreement with our proposed sequence of mutational events described in Bowles et al, 2007 [15]. Laurie et al., 2009, recently reported that loss of *Cdh11* correlated with optic nerve invasion using an *in vivo* model of *in vitro* cell lines derived from an *in vivo* murine retinoblastoma model [18].

In our present study, we use the TAg-RB retinoblastoma mouse model to study the function of *Cdh11* in tumorigenesis. This murine model, unlike any other RB mouse model, displays both molecular and histological features similar to the human disease [2], [11]–[13], [19]. Moreover, it is widely used as a pre-clinical model for testing therapeutics [14], [20]–[25]. Perhaps the strongest resemblance to human tumors is evidenced by its initiation in the inner nuclear layer (INL) of the retina and the presence of Flexner-Wintersteiner rosettes. The latter is an important feature not recapitulated in any of the other mouse models of retinoblastoma [26]–[30].

We now address the roles of *Cdh11* in developing retina and retinoblastoma. We report that *Cdh11* is developmentally regulated during retinogenesis. We show that *Cdh11* loss impacts the number of tumors that develop initially, and that it significantly increases the average tumor volume at PND84 per tumor initiating cell defined at PND8 in animals with mutant *Cdh11* alleles with respect to animals with wild type alleles. We also show clear *in vivo* and *in vitro* evidence that more cell death occurs in tumors with wild type alleles than with mutant *Cdh11* alleles, while cell proliferation remains unchanged regardless of *Cdh11* allele status. Taken together, these data provide substantial evidence to suggest that in retinoblastoma *Cdh11* acts as a tumor suppressor by facilitating cell death.

## Results

### Spatio-temporal expression and co-localization of cadherin-11 in the developing retina

**Figure 1:**
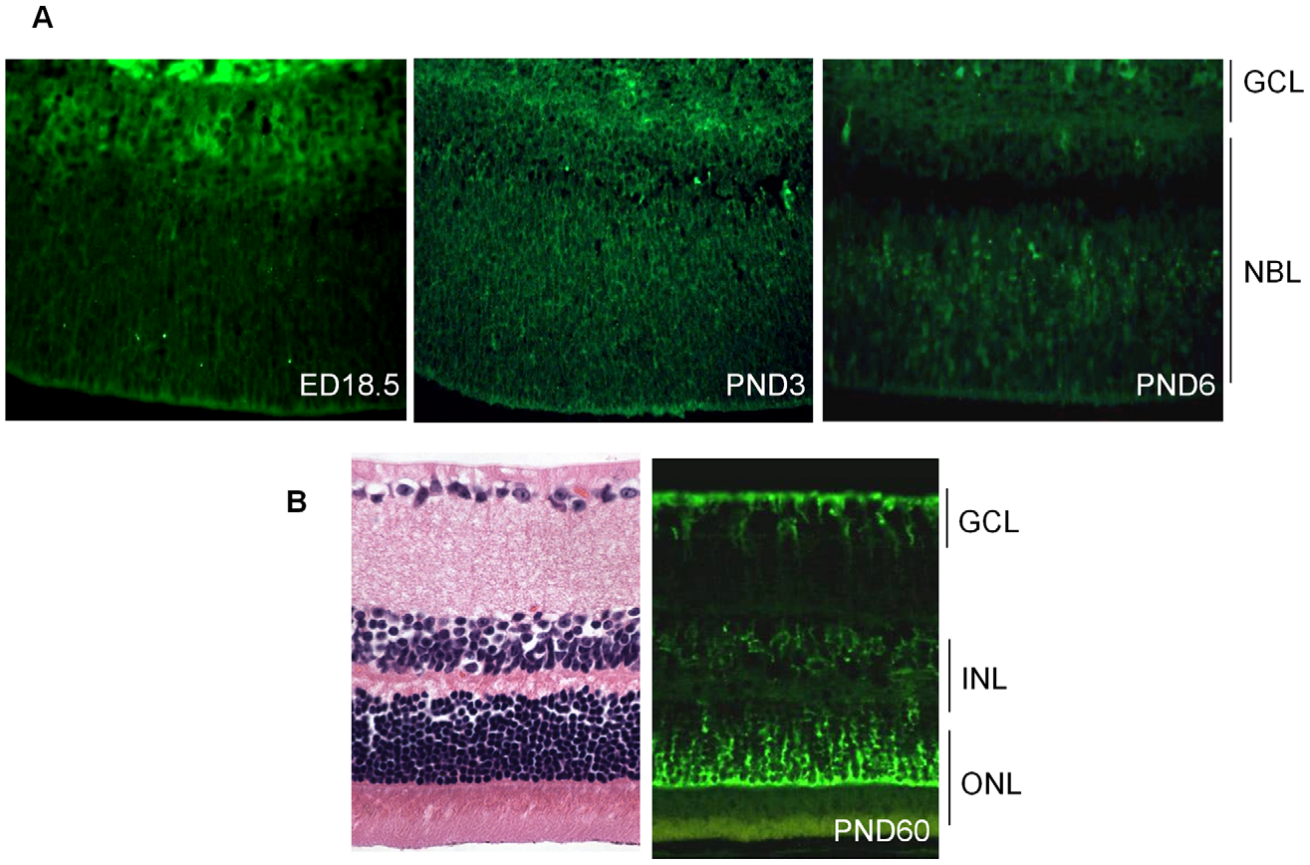
Expression of cadherin-11 in developing murine retina. (A) Cadherin-11 was expressed in the differentiating layer at (embryonic day) ED18.5, by retinoblasts at (post natal day) PND3 and again in a differentiating layer at PND6. (B) In adult mice, (PND60) cadherin-11 expression was restricted to cell types of the INL, with high expression by Müller glia processes that span the entire retina. GCL: ganglion cell layer; INL: inner cell layer; ONL outer nuclear layer.

To assess the role of *Cdh11* in the murine retina, we analyzed the spatio-temporal expression of cadherin-11 by immunostaining. Cadherin-11 was highly expressed by cells that typically differentiate at ED (embryonic day) 18.5 (Figure 1A). At PND3, expression was observed in areas where cells are migrating, and this became more visible in individual cells at PND6. At PND15 (data not shown) and adult (PND60), cadherin-11 was expressed in the inner nuclear layer (INL) by Müller glia cell bodies and processes at the outer border of the outer nuclear (ONL) and inner border of the ganglion cell layer (GCL) (Figure 1B).

**Figure 2:**
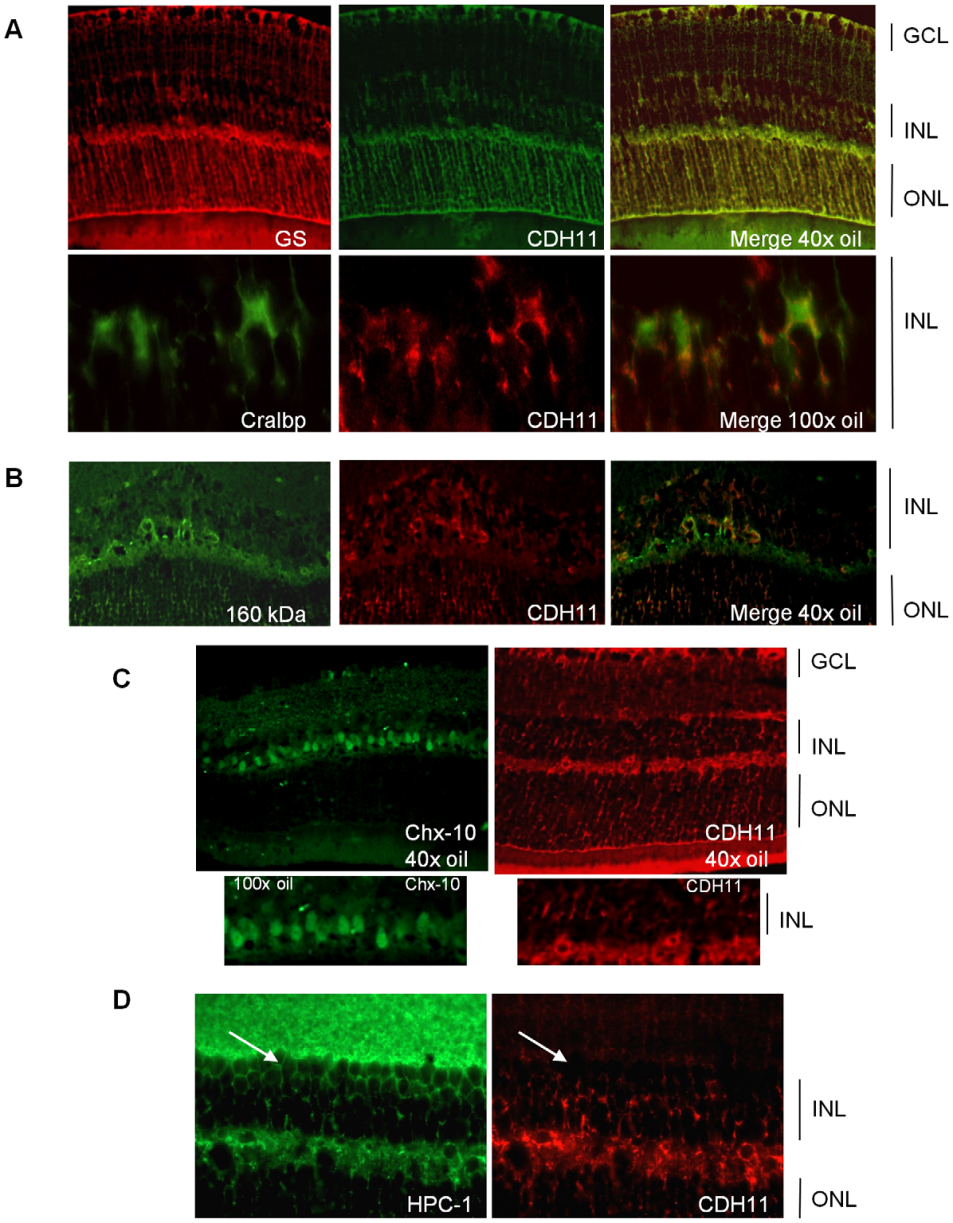
Co-expression of cadherin-11 and retinal cell types in adult retina. (A, B) Cadherin-11 expression co-localizes with Müller glia cell bodies (CRALBP, 100× magnification), Müller glia cell processes (glutamine synthetase, 40× magnification) and horizontal cells (160 kDa, 40× magnification) (C, D) but not with bipolar (Chx-10, 40× and 100× magnification) or amacrine (HPC-1, 40× magnification) (white arrows) cells.

To identify retinal cell types that express cadherin-11 in the INL, we performed co-localization studies in adult retina, using retinal specific cell type markers. Cadherin-11 co-expressed with markers of horizontal cells and Müller glia cells and their processes, (Figure 2A and 2B) but not with markers of bipolar or amacrine cells (Figure 2C and 2D).

### Retinal development in the absence of *Cdh11*

To examine the role of *Cdh11* in the developing retina, we studied littermates of *Cdh11* knockout animals. We analyzed retinas of *Cdh11^+/+^*, *Cdh11^+/-^*, and *Cdh11^-/-^* on a 129/C57Bl-6 mixed background at developmental time points ED18.5, PND3, PND6, PND15 and PND60. To accurately compare the retina of varying genotypes, retinal sections were cut every 5 µm throughout the eyes in the papillary-optic nerve plane.

**Figure 3:**
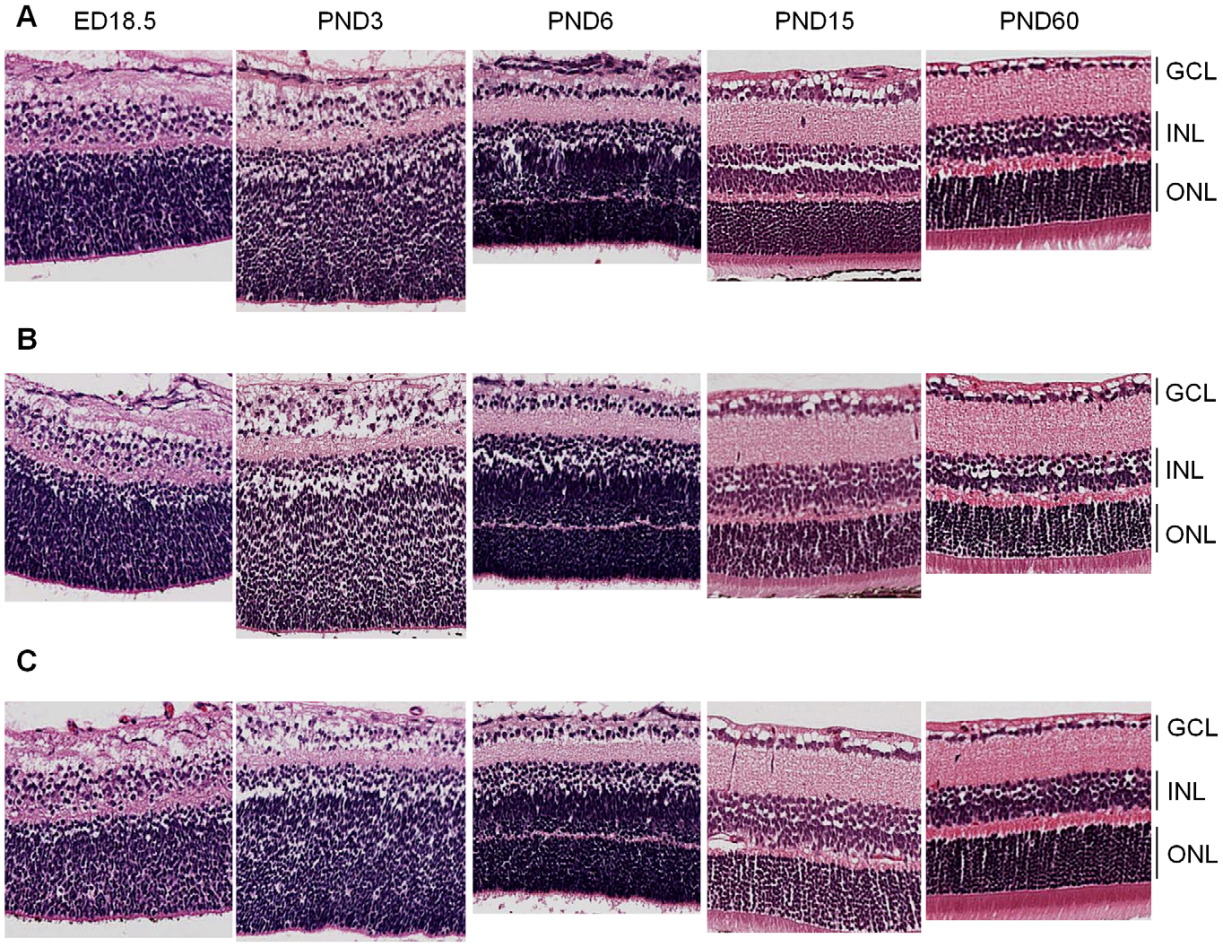
Retinal histology of *Cdh11* ^+/+^, *Cdh11* ^+/-^, and *Cdh11* ^-/-^ littermates. Hematoxylin and eosin (H&E) staining of 5 µm sections cut through the papillary-optic nerve plane. At developmental time points, ED18.5, PND3, PND6, PND15 (data not shown) and PND60 (adult), no gross retinal phenotypic differences were observed between (A) *Cdh11* ^+/+^, (B) *Cdh11* ^+/-^, and (C) *Cdh11* ^-/-^ littermates.

Hematoxylin and eosin (H&E) analysis of retinal sections at all developmental time points revealed no gross phenotypic differences between the *Cdh11* genotypes (Figure 3). Staining of retinal cell type markers was performed to determine if *Cdh11* influenced differentiation. There was no obvious change in cell populations that expressed Chx-10 (progenitor cells and bipolar cells), neurofilament (160 kDA for horizontal cells), cellular retinaldehyde-binding protein (CRALBP for Müller glia cells) or syntaxin (HPC-1 for amacrine cells) (Figure S1). The number of S-phase cells also seemed unaffected with loss of *Cdh11*, determined by immunohistochemical analysis of BrdU positive cells (Figure S1).

It is possible that the lack of gross phenotype in *Cdh11^-/-^* retinas is due to functional compensation by cadherins similar to *Cdh11*. *Cdh2*, also known as neuronal cadherin (N-cadherin), shares 53% amino acid similarity to *Cdh11* and is a mesenchymal cadherin like *Cdh11* [31]. However, immunohistochemical analysis showed no change in expression of *Cdh2* in the absence of *Cdh11* (Figure S1).

### Cadherin-11 expression in TAg-RB murine retinoblastoma tumors

**Figure 4:**
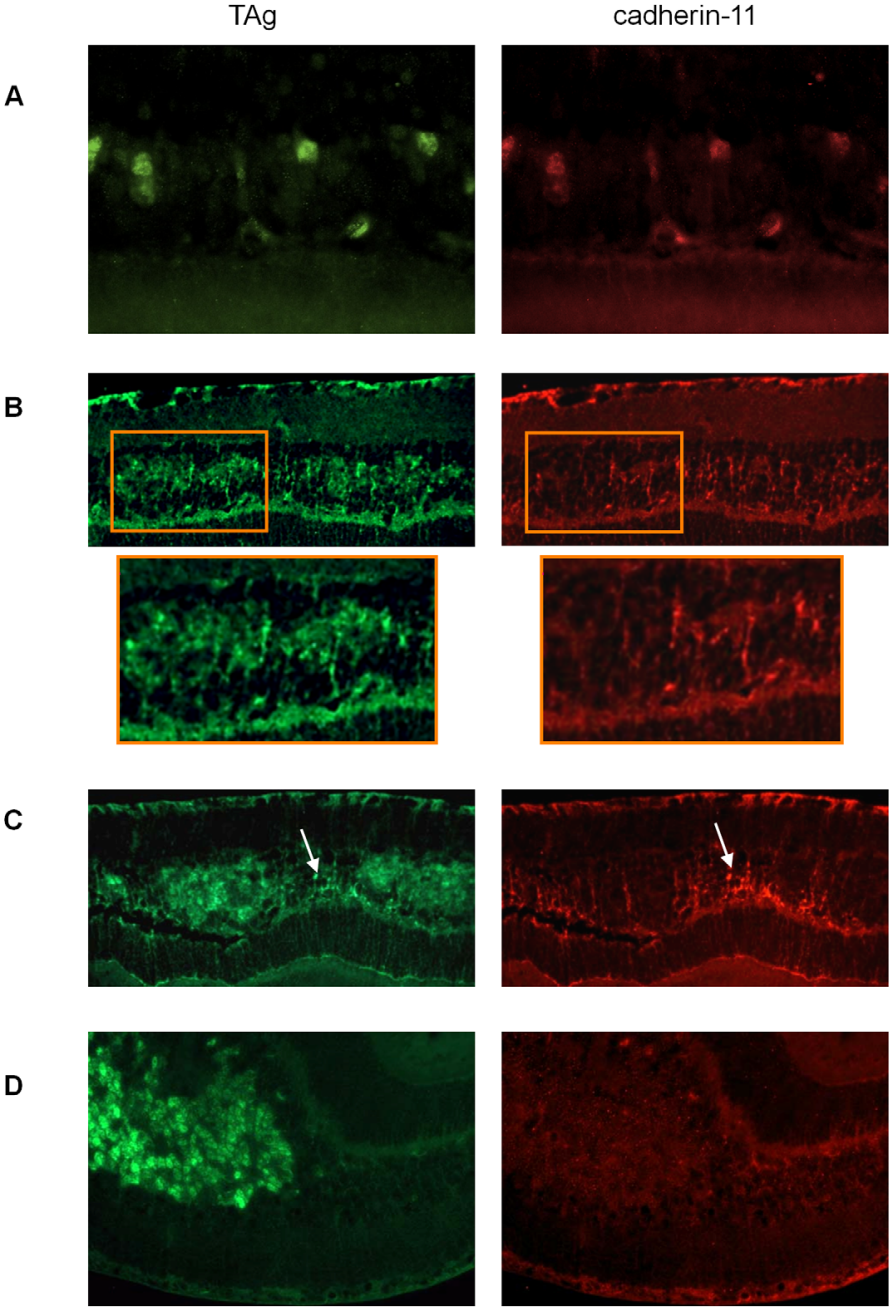
Gradual loss of cadherin-11 expression in TAg-RB tumors. (A) At PND9 TAg-RB mice displayed single TAg-positive cells (green) also positive for cadherin-11 (red). (B) At PND28 TAg-RB mice displayed multifocal tumors (clusters) which stained positive for TAg (green). Some of these multifocal tumors lost cadherin-11 expression (left cluster in box), while some retained expression (right cluster in box), suggesting a partial loss of cadherin-11 expression from PND28 tumors. (C) At PND35, regions of tumors that were positive for TAg were completely negative for cadherin-11 and adjacent normal cells retained cadherin-11 expression (arrow). (D) By PND140, entire tumors showed no cadherin-11 expression.

To evaluate cadherin-11 expression in developing tumors of the TAg-RB mouse model, we stained for cadherin-11 at PND9, PND28, PND35, PND84 and PND140. At PND9, early initiating tumour cells showed complete overlap of TAg and cadherin-11 staining (Figure 4A). At later time points, cadherin-11 expression was gradually lost from tumors: at PND28, some tumors showed loss and others showed expression (Figure 4B); at PND35, most tumors had lost expression of cadherin-11 (Figure 4C); by PND140, large, late stage tumors showed complete absence of cadherin-11 expression (Figure 4D).

### Tumor development in TAg-RB mice

**Figure 5:**
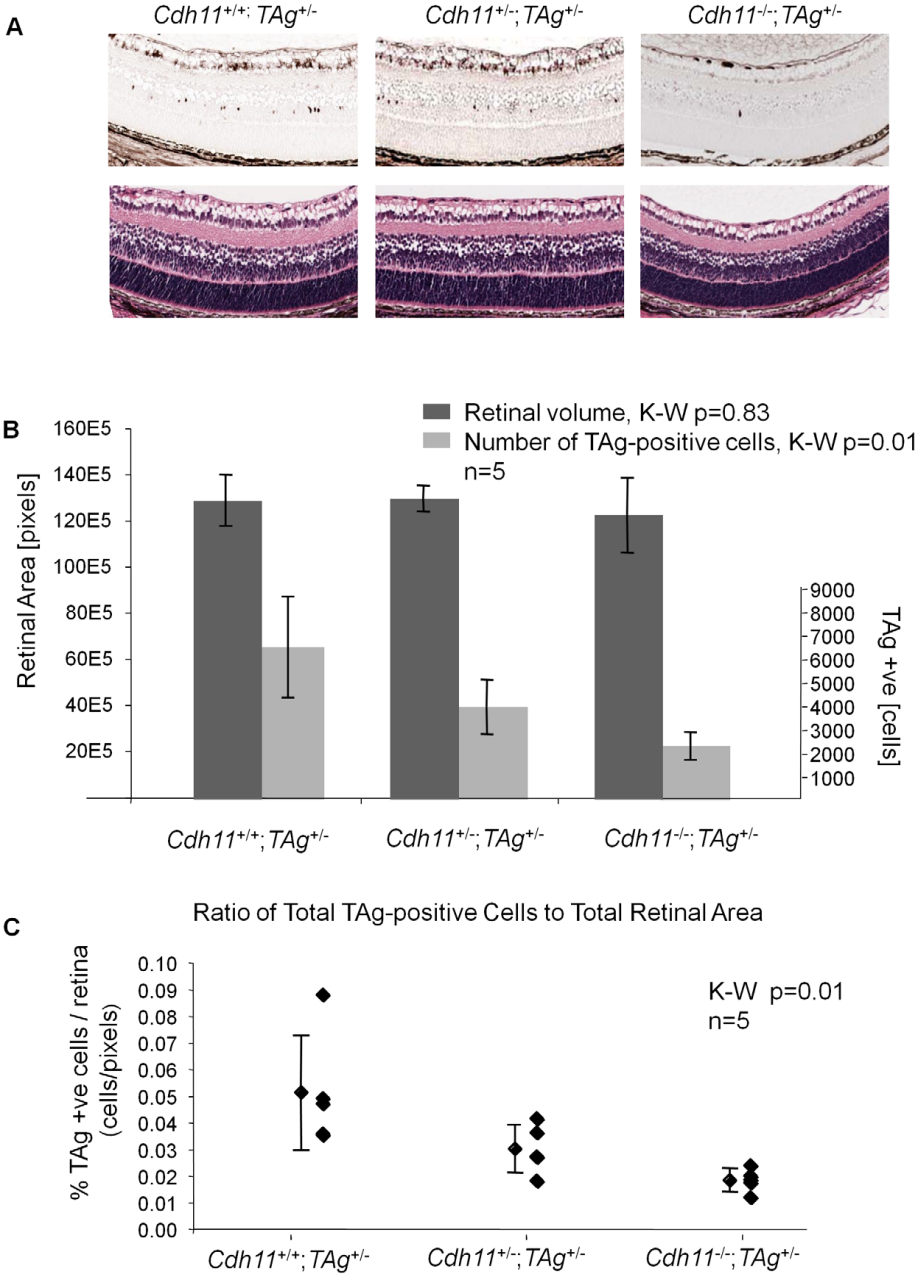
*Cdh11* genomic copy number correlates with number of TAg-positive cells (origin of tumors in TAg-RB mice) at PND8. (A) Representative sections of *Cdh11* ^+/+^;*TAg* ^+/-^, *Cdh11* ^+/-^;*TAg* ^+/-^, *and Cdh11^-^* ^/-^;*TAg* ^+/-^ genotypes by H&E stain and TAg staining. The single TAg-positive cells in the INL of the retina are reduced in number with reduced *Cdh11* allele dosage. H&E staining reveals no major phenotypic differences between the three genotypes. (B) Manual counts of TAg-positive cells per retinal area were extrapolated to the entire retina. The total number of TAg-positive cells of origin of retinoblastoma was 2-fold and 3-fold less (p = 0.01) (light grey bars) when one or two alleles of *Cdh11* were respectively lost, as compared to mice with normal *Cdh11*. The retinal size (dark grey bars) was similar (p = 0.83) between the *Cdh11* genotypes. (C) The ratio of TAg-positive cells to total retinal area was significantly reduced with reduced *Cdh11* gene dose (p = 0.01). The Kruskal-Wallis Test was used to assess difference between groups and error bars represent standard deviations.

**Figure 6:**
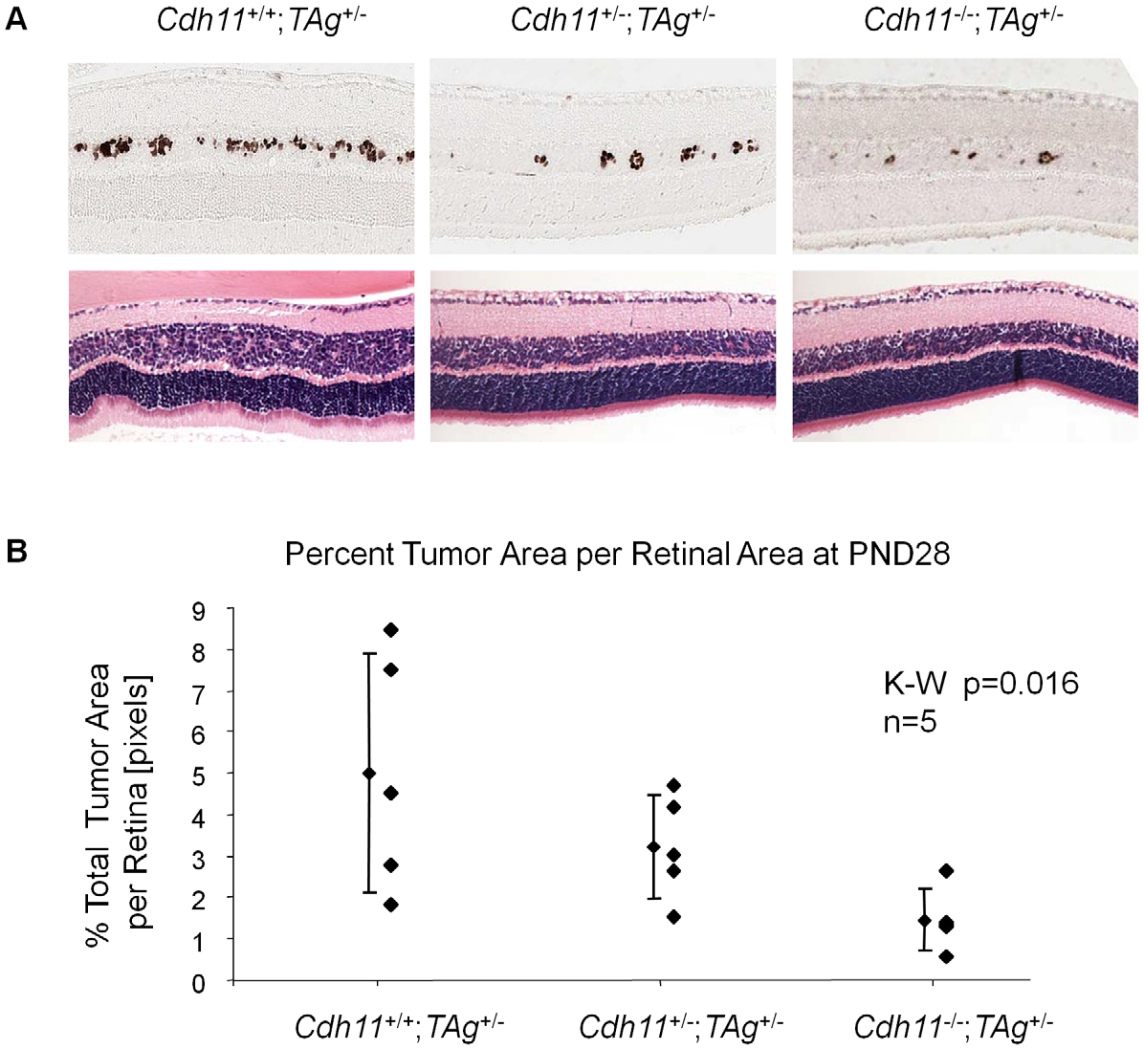
At PND28, fewer multifocal tumors developed when *Cdh11* alleles were lost. (A) A distinct *Cdh11* loss phenotype was observed from representative sections of TAg and H&E stains. Fewer TAg-positive multifocal tumors were present in mice with mutant *Cdh11* alleles; H&E showed more advanced tumors in *Cdh11* ^+/+^ mice than in *Cdh11* ^+/-^ or *Cdh11^-^* ^/-^ mice. (B) The number of multifocal tumors was significantly less (p = 0.016) in mice with *Cdh11* allelic loss, correlating with fewer tumor initiating cells at PND8. Total tumor volume was calculated using image J software measuring tumor area (TAg stained region) as a percentage of retinal area (manually traced) for every 60th section (approximately 300 µm apart) through the eye and extrapolated to the entire retina.

**Figure 7:**
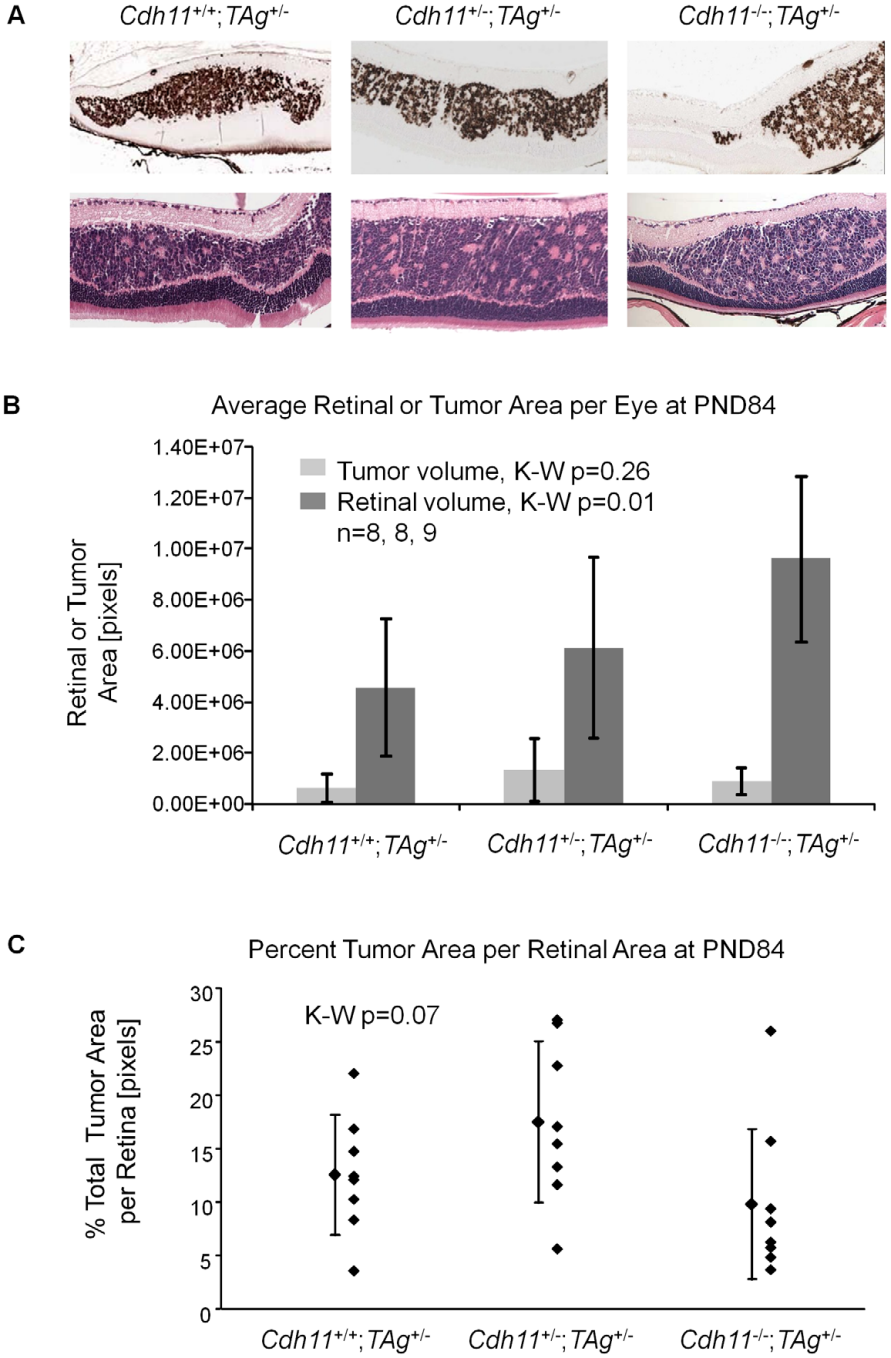
At PND84, total tumor volume was similar in all three genotypes. (A) Representative H&E and TAg stained sections showed large tumors originating from the INL of the retina. Tumors were composed of disorganized cells, rosette formations and disrupted laminated layers. No gross phenotypic differences were observed in different genotypes on H&E stained sections. (B) Retinal area and tumor area of every 60th section were tabulated and extrapolated to the entire retina. Total tumor volume per genotype was not statistically different (p = 0.26), but total retinal areas were significantly larger when *Cdh11* was lost (p = 0.01). (C) To accommodate for varying retinal size per genotype, total tumor volume was represented as a percentage total retinal area in all mice, showing no statistical difference between genotypes (p = 0.07), although a strong trend is observed, perhaps due to overall larger retinas. This suggested faster growing tumors in mice with *Cdh11* loss, since there were fewer tumor-originating cells and consequently fewer multifocal tumors initially (PND28). Tumor volume was calculated as described in Figure 5.

TAg-RB tumor development has been characterized (unpublished data). At PND8, TAg was first expressed by single cells in the INL of the retina (Figure 5A). At PND28, clusters of TAg-positive cells emerged (Figure 6A), consistent with multifocal tumors, each derived from single TAg expressing cells already present at PND8. These small tumor foci showed evidence of Homer Wright rosettes (data not shown). At PND84, tumors resembled human retinoblastoma (Figure 7A).

### Loss of *Cdh11* reduces the number of cells expressing TAg

To examine the tumor suppressor role of *Cdh11* in retinoblastoma development, we crossed *Cdh11^-/-^* mice with TAg-RB mice and analyzed genotypes *Cdh11^+/+^*;*TAg^+/-^*, *Cdh11^+/-^*;*TAg^+/-^*, and *Cdh11^-/-^*;*TAg^+/-^*, on a mixed 129/C57Bl-6 background. Gross phenotypes at varying time points were assessed by H&E staining.

At PND8, retinal histology of mice with normal and *Cdh11* allelic losses showed no differences in H&E staining (Figure 5A). Immunostaining showed that TAg was expressed by large, spindle shaped single cells in the INL (Figure 5A). Tissue sections taken every 300 µm spanning the entire eye were manually counted for TAg-positive cells and the total number of TAg-positive cells per eye was extrapolated to the entire retina based on the total number of sections that were produced per eye (Figure 5B, for detailed report of this method see [32]). A striking reduction in the number of TAg-positive cells was observed in retinas of mice with mutant *Cdh11* alleles compared to mice with normal *Cdh11* alleles. Animals of the *Cdh11^+/+^*;*TAg^+/-^* genotype had a mean of 6,417 TAg-positive cells per entire retina compared to 3,874 and 2,230 in *Cdh11^+/-^*;*TAg^+/-^* and *Cdh11^-/-^*;*TAg^+/-^* genotypes respectively, describing a significant allele dosage effect (p = 0.01, n = 5) (Figure 5B). As a control and to normalize the total TAg-positive cells per retina, retinal area was measured for each of the selected sections, using the *Image J* software and then extrapolated to the entire retina. Total retinal areas at PND8 were found to be similar in all *Cdh11* genotypes (p = 0.83, n = 5) (Figure 5B). To quantify tumor-initiating cells with respect to retinal area, we determined the *ratio* of TAg-positive cells per retinal area, which showed a significant reduction correlated with *Cdh11* genomic loss (p = 0.01) (Figure 5C). This effect continued at later stages in development, since at PND28, fewer multifocal tumors developed in mice with *Cdh11* loss. These data suggest that in this model, the expression of TAg may be dependent on *Cdh11*.

### Evaluating tumor volume at PND28 and PND84

At PND28, we observed a significant decrease in the number of multifocal tumors with decreasing number of functional copies of *Cdh11* as assessed by both H&E and TAg stain (Figure 6A). Tumor volumes as a percent of retina were estimated to be 5.0%, 3.2% and 1.5% in *Cdh11^+/+^*;*TAg^+/-^*, *Cdh11^+/-^*;*TAg^+/-^* and *Cdh11^-/-^*;*TAg^+/-^* genotypes respectively (5 animals analyzed per genotype). These analyses describe a significant decrease in tumor volume as *Cdh11* alleles are lost (p = 0.016, Figure 6B).

At PND84, tumor morphology of the varying genotypes did not differ by H&E or TAg staining (Figure 7A). All three genotypes showed tumors highly reminiscent of human retinoblastoma, presenting with large tumors originating from the INL, bulging into adjacent layers, and displaying features of Homer Wright rosettes (Figure 7A).

In stark contrast to earlier timepoints, total tumor volume at PND84 was not significantly different in mice of *Cdh11^+/+^*;*TAg^+/-^*, *Cdh11^+/-^*;*TAg^+/-^* and *Cdh11^-/-^*;*TAg^+/-^* genotypes (p = 0.26; n = 8, 8, and 9 respectively, Figure 7B). However, unlike in the younger mice, total retinal size was significantly larger (p = 0.01) in the *Cdh11* null mice compared to *Cdh11* normal mice (Figure 7B), suggesting that loss of *Cdh11* may affect the overall size of the adult retina in TAg mice. Tumor volume as a percentage of the entire retina was not significantly different between genotypes (p = 0.07, Figure 7C). The similarity of tumor volume at PND84 suggests faster tumor growth may be occurring in mice with mutant *Cdh11* alleles, considering that fewer multifocal tumors were initially present at PND28. These data suggest two roles for *Cdh11* in retina: 1) *Cdh11* displays tumor suppressor abilities *in vivo* and 2) *Cdh11* loss affects retinal development in TAg mice, reflected in increase in overall size of the adult retina. This difference was not observed up to PND60 in *Cdh11^-/-^* mice (Figure 3).

### Faster tumor growth is observed from PND8 to PND84 in mice with mutated *Cdh11* alleles

**Figure 8:**
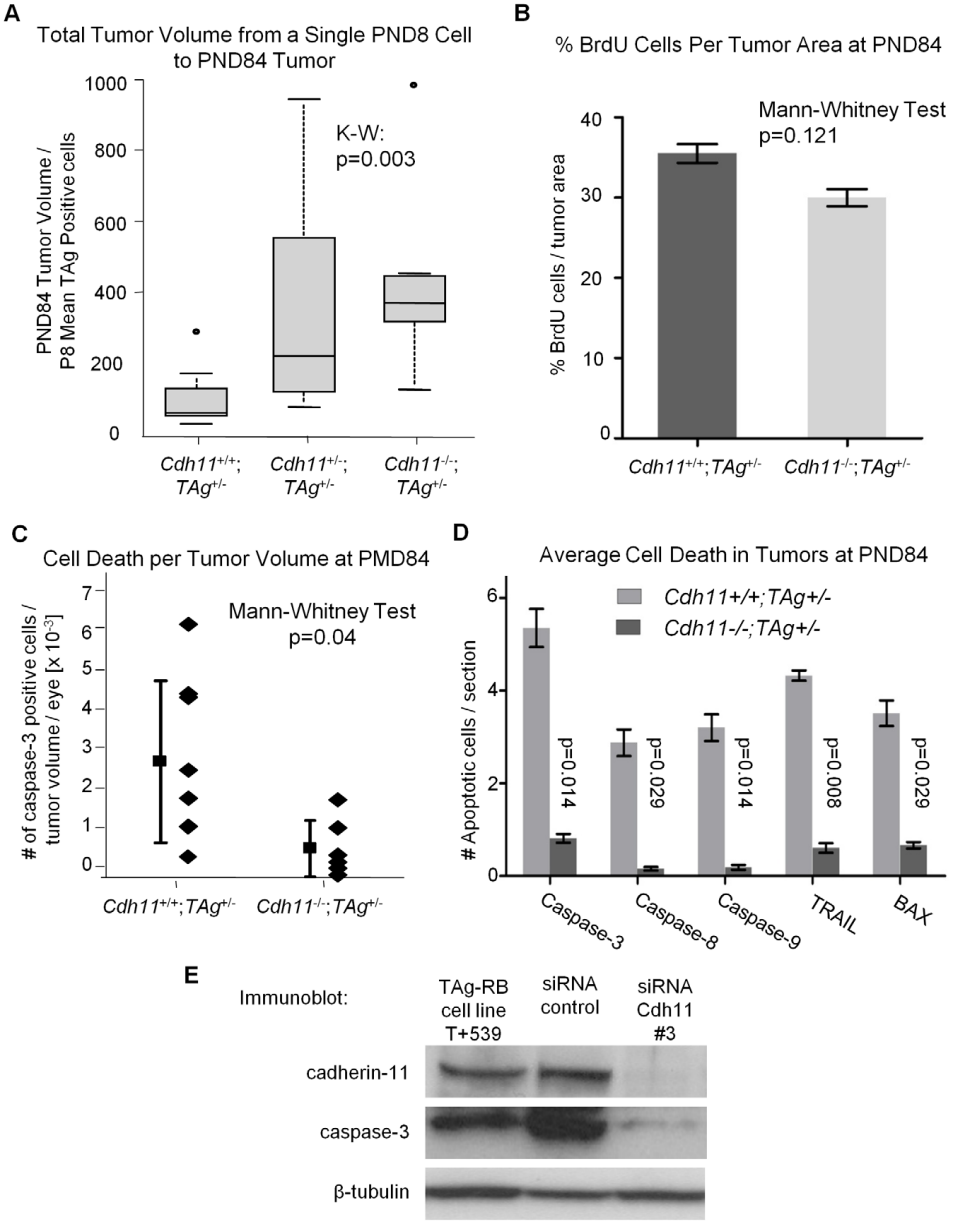
Allelic loss of *Cdh11* led to faster growing tumors due to decreased cell death. (A) The number of single tumor initiating cells at PND8 was estimated by averaging the total number of TAg-positive cells in 5 mice per genotype. The ratio of tumor volume in pixels at PND84 to the average number of TAg-positive cells per eye at PND8 for the three genotypes was used to estimate tumor growth rate. A significant difference in growth rate between groups was observed (p = 0.003), with a 3-fold increase between *Cdh11* ^-/-^ and wild type mice. After controlling for larger retinas (Figure 6B), growth rate remained significantly larger in *Cdh11* ^-/-^ mice (data not shown). (B) *Cdh11^+/+^;TAg^+/-^* and *Cdh11^-/-^;TAg^+/-^* PND84 eyes were stained for BrdU incorporation (n = 6 per group). Proliferation was analyzed by averaging slides for the % BrdU positive cells (in pixels) per tumor area (determined by TAg staining in pixels). No significant difference was observed (p = 0.121). (C) Every 60th section of *Cdh11^+/+^*;*TAg^+/-^* (n = 8) and *Cdh11^-/-^*;*TAg^+/-^* (n = 6) PND84 eyes, was counted for tumor cells positive for activated caspase-3, extrapolated to the entire retina and represented as a ratio to tumor volume per eye. The number of dying cells in tumors of mice with normal *Cdh11* alleles are significantly more abundant (p = 0.04) than in tumors of mice with mutant *Cdh11* alleles. (D) To further support this data, we analyzed for the expression of five pro-apoptotic proteins (activated caspase-3, 8, 9, Trail and Bax) in an additional cohort of *Cdh11^+/+^;TAg^+/-^* and *Cdh11^-/-^;TAg^+/-^* PND84 animals. Staining and analysis were performed as performed previously. Results revealed between five to ten times less apoptotic activity in TAg-RB tumors null for *Cdh11* (p = 0.014, 0.029, 0.014, 0.008, and 0.029, respectively; n = 4 per group). (E) Cadherin-11 was knocked down using stealth siRNA in a cadherin-11 expressing cell line derived from TAg-RB tumors, T +539. Knockdown of *Cdh11* clearly decreased caspase-3 expression compared to control.

To establish whether tumors developing in *Cdh11* mutant animals grew faster, we studied the rate of tumor growth between PND8 and PND84. This was done by calculating the ratio of tumor volume at PND84 (in pixels) to the mean number of TAg-positive cells (single tumor initiating cells) at PND8. The analysis revealed significant differences between the genotypes (p = 0.003, Figure 8A), indicative of faster growing tumors in mice with mutant *Cdh11* alleles. We performed a second comparison to account for the difference in retinal size between genotypes at PND84 by calculating the ratio of percent tumor volume per retina at PND84 to the mean number of TAg-positive cells in the entire PND8 retina per genotype. Even after adjusting for retinal size, the tumor volume per initiating cell in mice with mutant *Cdh11* alleles remained significantly greater (p = 0.01, data not shown). In addition, we noticed that while the retinal size at PND8 was similar between genotypes (p = 0.83, Figure 5B), the PND84 retinal size was significantly larger (p = 0.01, Figure 7B), suggesting a role for *Cdh11* in the retinal development of TAg-RB mice.

### *Cdh11* mediates its tumor suppressor function through apoptosis and not proliferation

Since “growth” reflects a positive balance between cell proliferation and cell death we evaluated both cell proliferation and death in tumors at PND84.

At PND84, tumors are well defined and easily quantifiable. We performed PCNA staining (a marker of cells in early G1 and S phase) of PND84 tumors in selected sections of *Cdh11^+/+^*;*TAg^+/-^* (n = 2) and *Cdh11^-/-^*; *TAg^+/-^* (n = 2) mice and calculated the percent PCNA positive cells per tumor volume revealing little difference between the two genotypes. To improve the power of this analysis, BrdU incorporation in PND84 tumors was evaluated in an additional larger cohort of animals. Again, we noticed no significant difference between the genotypes (p = 0.121, n = 6 for each genotype, Figure 8B). These data strongly support that *Cdh11* is not acting to impede proliferation of tumor cells.

To assess cell death, selected sections of *Cdh11^+/+^*;*TAg^+/-^* (n = 8) and *Cdh11^-/-^*; *TAg^+/-^* (n = 6) were manually counted for activated caspase-3 positive cells per tumor area and extrapolated to the entire tumor volume. Non-tumor retina showed no activated caspase-3 positive cells. We found significantly more cell death in tumors of mice with normal *Cdh11* alleles than in tumors of mice with mutated *Cdh11* alleles (p = 0.04, Figure 8C). Interestingly, β-catenin mRNA was upregulated in the *Cdh11^-/-^ TAg^+/-^* mice relative to the *Cdh11^+/+^Tag^+/-^* mice (Figure S2C).

Furthermore, we observed a wide distribution of cell death among *Cdh11^+/+^*;*TAg^+/-^* mice (mean = 2.90×10^−03^, standard deviation ±2.08×10^−03^) compared to mice with mutant *Cdh11* alleles (mean = 6.94×10^−04^, standard deviation ±7.25×10^−04^, Figure 8C). To further support the role of *Cdh11* in apoptosis, we assayed by immunohistochemistry, in an additional cohort of animals, five pro-apoptotic proteins: activated caspases 3, 8, 9, TRAIL and BAX. Depending on the cell death marker, we observed 5 to 10 fold less expression in *CDH11* mutant animals than in animals with normal *Cdh11* alleles (p<0.03 for all five cell death markers, Figure 8C).

We also assessed cell death *in vitro* in a primary cell line derived from TAg-RB tumors (T+539). This tumor cell line, when treated with cadherin-11 siRNA, showed significant cadherin-11 knockdown (Figure 8D). Following knockdown, caspase-3 expression was decreased (Figure 8D), providing further evidence that *Cdh11* acts to promote apoptosis. In addition, we studied RNA from the T+539 cell line treated either with *Cdh11* siRNA or scrambled siRNA by RT-PCR for proliferation markers PCNA and Ki67, and found no difference in expression (Figure S3). These data strongly support the hypothesis that *Cdh11* has a pro-apoptotic role in TAg-RB tumors, but does not suggest a role in cell division or proliferation.

## Discussion

### *Cdh11* displays tumor suppressor-like properties *in vivo*

We have previously described copy number and expression loss of *CDH11* in human retinoblastomas, suggesting a tumor suppressor role [13]. We now confirm the tumor suppressor role *Cdh11* in retinoblastoma through functional experiments. The 97kD *Cdh11* isoform that is retained in the *Cdh11* knockout model we studied, has been documented to lack adhesion properties and thus likely represents a non-functional protein [33], [34]. By crossing this *CDH11* functional knockout with the TAg-RB mice, we report an unexpected result: *Cdh11* allelic loss results in fewer tumor initiating TAg positive cells at PND8 (Figure 5), and consequently fewer multifocal tumors at PND28 (Figure 6) compared to animals with normal *Cdh11* alleles. This suggests that TAg transgene expression may be affected by the loss of *Cdh11* (Figure 5).

Loss of *Cdh11* in *Cdh11^-/-^* mice did not affect retinal size up to PND60 (Figure 3). At PND8 retinal volumes were similar in *Cdh11* ^+/+^;*TAg* ^+/-^, *Cdh11* ^+/+^;*TAg* ^+/-^, and *Cdh11* ^+/+^;*TAg* ^+/-^ mice, but at PND84, the total retinal size was significantly larger (p = 0.01) in the *Cdh11^-/-^;TAg^+/-^* mice compared to *Cdh11^+/+^*;T*Ag^+/-^* mice (Figure 7B), suggesting that loss of *Cdh11*, when combined with the expression of TAg, affects the overall size of the adult retina in TAg-RB mice. Our previous studies of the *Cdh11^-/-^* retina, quantifying the individual retinal cell types visualized by immunofluorescence with cell-specific antibodies, showed no difference between the *Cdh11^-/-^* and wild type retina [35].

At PND84, we show that absolute tumor volume was not statistically different between all three genotypes (total tumor volume alone or as a percentage of the retinal volume). However, since these tumors arise from fewer tumor-initiating cells, we conclude that tumor growth per initiating cell was greater in mice with mutant *Cdh11* alleles (Figure 7B and 7C, Figure 8A). We conclude that *Cdh11* functions as a tumor suppressor. Since tumor “growth” results from an imbalance between cell death and proliferation, we examined cell proliferation (Figure 8B) and cell death (Figure 8C) in TAg-RB tumors of mice with normal *Cdh11* alleles versus mutated *Cdh11* alleles. Our data indicate that when *Cdh11* is lost, cell death is deficient while proliferation remains unchanged, suggesting that the tumor suppressor function of *Cdh11* is mediated through promotion of apoptosis rather than inhibition of cell proliferation. This is further supported by our *in vitro* data showing significant decrease in caspase-3 and increase in β-catenin expression in *Cdh11* knockdown experiments using siRNA (Figure 8D and Figure S2A, S2B), while proliferation markers PCNA and Ki67 remain unchanged (Figure S3).

The spread of tumor volumes across the various time points is narrowed in mice that have lost both *Cdh11* alleles. We speculate that tumors in mice with normal *Cdh11* alleles could be losing functional *Cdh11* at varying timepoints during tumor development, and the wide spread in tumor volume reflects heterogeneity for *Cdh11*. In contrast, mice with both *Cdh11* alleles mutated have more consistent measures of cell death (Figure 8C). This agrees with our previous report where some tumors display loss of *Cdh11*, while others retain it at later timepoints [13]. In summary, we describe a mechanism by which *Cdh11* may be functioning as a tumor suppressor gene in retinoblastoma.

Additional experiments need to be performed to assess the mechanism by which *Cdh11* facilitates cell death in these tumors. Our preliminary experiments have shown increased protein and mRNA levels of β-catenin when *Cdh11* is knocked down, and increased β-catenin mRNA in PND84 *Cdh11^-/-^TAg^+/-^* mice relative to *Cdh11^+/+^TAg^+/-^* mice (Figure S2A, S2B, S2C). Upon cell-cell contact, cadherin molecules form the adherens junction. The cadherin binds directly to β-catenin, which recruits α-catenin to link the complex to the cytoskeleton. This is necessary to maintain cell-cell adhesion and cellular architecture [36]. These junctions are dynamic and the structure and signaling provided by the complex ultimately determines the cellular phenotype and behavior [37]. β-catenin is additionally a major regulator of the Wnt signaling pathway. The Wnt-signaling pathway is implicated in other cancers [38], [39] and suppresses apoptosis through both β-catenin dependent and independent pathways [40]. Many studies have shown that cadherin protein levels impact canonical Wnt-signaling and β-catenin levels. Gain and loss of function studies support cadherins directly sequestering β-catenin from the nucleus, acting as a sink for the cytosolic pool [41]–[43]. Additionally, downregulation of E-cadherin expression has been paralleled with an upregulation of β-catenin in hepatocellular carcinoma tumors [44]. Next investigations would test the possibility that down regulation of cadherin-11 affects the levels of canonical Wnt signaling in these TAg-RB cell lines, that may lead to the decrease in cell death and faster growing tumors.

### *Cdh11* supports the tumor initiating cell in the TAg-RB mouse model

Previous studies of cell adhesion molecules in the neural retina have described that expression of cadherin subtypes is restricted to different retinal cell populations. Based on these studies the authors suggested that cadherins play a role in maintaining selective neuronal associations [45], [46]. In order to understand the role of *Cdh11* in retinoblastoma progression, we examined its presence during healthy retinal development.

We showed that *Cdh11* is developmentally regulated. Expression was restricted to differentiating/migrating retinal cells at E18.5 through to PND6, and to the INL at PND60 (adult) (Figure 1). Cadherin-11 co-expresses with markers of Müller glia cell bodies and processes that span the entire retina (Figure 1B and Figure 2B). Prominent expression of cadherin-11 by retinoblasts at PND3 and PND6 in the developing retina and co-expression with Müller glia and horizontal cell types, suggests roles for cadherin-11 in morphogenesis, such as cell migration, sorting or positioning of these cells (Figure 1A) during retinal development.

The tumor -initiating cell in the TAg-RB mouse model has been identified to belong to a subset of the Müller glia (unpublished data). Our results indicate that when *Cdh11* alleles are mutated in TAg-RB mice, fewer cells express TAg and develop into retinoblastoma. It is possible that *Cdh11* loss affects the expression of the TAg transgene in this murine model, or that it affects development of the subpopulation of Müller glia that gives rise to the TAg-RB tumours. We were unable to discern the latter, since *Cdh11* ^-/-^ mice do not show a significant change in retinal cell type distribution in the retina, and so few of this retinal subtype express TAg in this model (unpublished data). From these data, we suggest that *Cdh11* has an important role in the expression of TAg from the transgene in this murine model.

### Summary and significance

We describe the use of the retinoblastoma TAg-RB mouse model to study specific gene function in tumor development. This was achieved by crossing TAg-RB mice to *Cdh11^-/-^* mice. We showed that *Cdh11* is a suppressor of retinoblastoma progression by using a unique and highly sensitive method to identify and quantify tumor volume. Although fewer multifocal tumors initiate in mice with mutant *Cdh11* alleles, suggesting that *Cdh11* loss modulates the number of TAg-espressing cells in this murine model, the resulting tumors grow faster, describing a tumor suppressor role for *Cdh11* in retinoblastoma progression. Significantly reduced numbers of cells stained for pro-apoptotic proteins in tumors of mice with absent *Cdh11* alleles, indicating that promotion of cell death is an important part of the tumor suppressor action of *Cdh11*.

## Materials and Methods

### Animals

All animals were maintained and sacrificed using protocols approved by the Animal Care Committee of the Ontario Cancer Institute (OCI) which adhere to the EC Directive 86/609/EEC for animal experiments.

*Cdh11^-/-^* mice, background strain 129, were provided by Dr. M. Takeichi [33]. To study the role of *Cdh11* in retinal development, one-generation crosses were made between *Cdh11^-/-^* 129 and *Cdh11^+/+^* C57-Bl-6 to get a mixed background of 129/C57Bl-6. Littermates, *Cdh11^+/+^*, *Cdh11^+/-^* and *Cdh11^-/-^* on this 129/C57Bl-6, mixed background were sacrificed at developmental time points: embryonic day (ED)18.5, post-natal day (PND)3, PND6, PND15 and PND60. To analyze proliferating cells, pregnant mothers at ED18.5, pups at PND3 and PND6, and adults at PND84 were injected with bromodeoxyuridine (BrdU) reagent (5-bromo-2′-deoxyuridine and 5-fluoro-2′-deoxyuridine, 10∶1, used at 1 ml reagent per 100 g body weight, Cat# 00-0103, Lot# 60203722, Zymed Laboratories) for 2 hours and then sacrificed.

TAg-RB (*TAg^+/-^*), background strain C57/Bl-6, mice were provided as a gift from Joan O'Brien [16]. One generation crosses were made between *Cdh11^-/-^* and *TAg^+/-^* mice to get double heterozygotes, *Cdh11^+/-^*; *TAg^+/-^*, on a 129/C56/Bl-6 mixed background. Mice were further crossed with *Cdh11^-/-^*, *Cdh11^+/-^* or *Cdh11^+/+^* of a 129/C56Bl-6 mixed background to get the three genotypes analyzed for this study: *Cdh11^+/+^*;*TAg^+/-^*, *Cdh11^+/-^*;*TAg^+/-^* and *Cdh11^-/-^*;*TAg^+/-^*. These animals were sacrificed at PND8, PND28 and PND84, the latest time point we could study in compliance with our Animal Protocol at the Ontario Cancer Institute.

Genotyping of *Cdh11^-/-^* mice and littermates were carried out using PCR conditions: 94°C, 2 min, 1 cycle, [94°C, 30 sec, 50°C, 30 sec, 72°C 30 sec] 30 cycles, 72°C 10 min, and 4°C cool block. Primers used were: forward, 5′ to 3′ (21 bp): ttc agt cgg cag aag cag gac and backward, 5′ to 3′ (19 bp): gtg tat tgg ttg cac cat g, and neo, 5′ to 3′ (23 bp): tct atc gcc ttc ttg acg agt tc. Sizes of expected PCR products were: *Cdh11^+/+^*: 240 bp, *Cdh11^+/-^*: 480 bp and 240 bp, and *Cdh11^-/-^*: 480 bp. Genotyping of *TAg^+/-^* mice and their littermates were carried out using similar PCR conditions: 94°C, 2 min, 1cycle, [94°C, 1 min 58°C, 1 min, 72°C 1 min] 30 cycles, 72°C 10 min, 1 cycle and 4°C cool block. Primers used were: forward 5′ to 3′: gac ttt gga ggc ttc tgg gat gca act gag and backward 5′ to 3′: ggc att cca cca ctg ctc cca ttc atc agt. Size of expected PCR product was 420 bp.

### Histology and slide selection

Heads and/or eyes were fixed in freshly prepared 4% PFA/PBS for 48 hrs and then stored in 70% Ethanol. Heads were decalcified (8% formic acid following 4% PFA) for approximately 1 week. Both heads and/or eyes were paraffin embedded and 5 µm sectioned.

For *Cdh11^+/+^*, *Cdh11^+/-^* and *Cdh11^-/-^* littermates: Serial sections were made specifically through the papillary-optic nerve plane (approx. 20 sections in total) for consistent comparison between genotypes.

For *Cdh11^+/+^*;*TAg^+/-^*, *Cdh11^+/-^*;*TAg^+/-^* and *Cdh11^-/-^*;*TAg^+/-^* mice: Serial sections were made through the entire eye (approximately 270–420 sections per eye with 5–7 sections made per slide). To estimate tumor volume per eye, we selected one slide every 60th section (approx. one slide every 300 µm) for analysis. A total of about 5–8 slides were analyzed per eye. Only one eye was analyzed per mouse.

### Immunohistochemistry

Slides selected for analysis were studied using the immunohistochemical protocol described previously[12]. Briefly, slides were incubated with primary, then biotinylated secondary antibodies, either anti-mouse, anti-rabbit, anti-goat, or anti-sheep, used at a dilution of 1∶200 with 10% DakoCytomation Antibody Diluent in 1% BSA/TBST for 1 hr at room temperature. To visualize TAg, BrdU and Brn3b (ganglion) stained cells, we employed an Immunopure DAB Substrate Kit (Cat# 34065, Pierce). After incubation with primary and biotinylated secondary antibodies, slides were incubated for 1 hr at room temperature in an ABC prepared solution (Vectastain ABC Elite, Vector Laboratories). Stained cells could be visualized after a maximum of half an hour incubation in DAB substrate solution (Pierce) prepared fresh with 10% DAB/Metal Concentrate, 10× (Product# 1856090) made in Stable Peroxide Substrate Buffer, 1× (Product# 1855910). All other proteins were visualized by immunofluorescence; after incubation with primary and secondary antibodies, slides were washed in 1×TBS and then incubated with Streptavidin-Alexa488 or Streptavidin-Alexa594, used at 1∶200, prepared in 1×TBS for 15 min at room temperature. Slides were washed briefly in 1×TBS and incubated in 4′, 6-diamino-2-phenylindole (DAPI) used at 1∶50, followed by wash in 1×TBS and mouniting with DakoCytomation Fluorescent Mounting Medium (S3023). Selected slides were Haematoxylin and eosin (H&E) stained for light microscopy analysis. Table 1 provides a complete list of all antibodies used. Antibodies to recognize specific cell types were: Hes-5 [47], CRALBP [48] and glutamine synthetase [49] (early Müller glia, Müller glia cell bodies and processes), syntaxin [50] (HPC-1 for amacrine cells), neurofilament 160kDa [51] (horizontal cells), Brn3b [52] (ganglion cells) and Chx-10[53] (bipolar cells).

**Table 1:** Antibody list.

Antibody Name	Company	Dilution for IHC
SV40 TAg (Pab 101)	Santa Cruz Biotechnology	1∶200
mouse monoclonal	Cat# SC-147, Lot# A2506	
CDH11 - clone CDH113H	Gift from Dr. St. John	1∶2500
mouse monoclonal	at ICOS Corp.	
CDH2 (N-cadherin)	BD Biosciences Pharmigen	1∶2000
mouse monoclonal	Cat# 610920, Lot# 06247	
BrdU (purified anti-bromodeoxyuridine)	BD Biosciences Pharmigen	1∶200
mouse monoclonal	Cat# 555627, Lot#52817	
Progenitors and Bipolars: Chx-10 sheep polycolonal	Gift from Rod Bremner, UHN	1∶1000
Early Müller Glia: Hes-5	ABCAM	1∶50
rabbit polyclonal	Cat# AB25374	
Müller Glia: CRALBP; rabbit polyclonal	Gift from John Saari	1∶6000
Müller Glia: Vimentin	Santa Cruz Biotechnology	1∶100
goat polyclonal	Cat#SC-7557	
Ganglion: Brn3b	Santa Cruz Biotechnology	1∶100
goat polyclonal	Cat# SC-6026	
Amacrine: Syntaxin clone, HPC-1	Sigma	1∶200
mouse monoclonal	Cat# S0664	
Horizontal: Neurofilament 160 kDa	Sigma	1∶40
mouse monoclonal	Cat# N5264	
TRAIL	Abcam	1∶100
goat polyclonal	Cat# SC-6079	
BAX	Santa Cruz Biotechnology	1∶100
goat polyclonal	Cat# SC-526	
Activated Caspase-8	Abnova	1∶1000
rabbit polyclonal	Cat# PAB0246	
Cleaved Caspase-9	Cell Signaling Technology	1∶100
rabbit polyclonal	Cat# 9509	
Activated Caspase-3	R&D Systems	1∶500
rabbit polyclonal	Cat# AF835, Lot# CFZ326011	

This table describes all antibodies used in this study.

### Image analysis and quantification of tumor volume in mouse retina

Of the techniques described to measure tumor volume in murine retinoblastoma, none are useful to quantify small, developing tumors at very early time points [20]–[22],[24],[25]. Therefore, we developed a novel technique to quantitate tumor volume in the eyes of TAg-RB mice by analyzing every 60th section through the entire eye [32]. Tumor development was tracked by staining for TAg. Diamino benzidine (DAB) typically stains TAg cells brown with very little background, however in some cases, background staining is visible in the GCL and retinal pigment epithelium (Figure 4, Figure 5, and Figure 6). Total tumor area per eye was quantified as a percentage of total retinal area (measured in pixels) using Image J software. The selected sections were scanned at the Advanced Optical Microscopy Facility at the Ontario Cancer Institute using an Aperio ScanScope CS. Images were retrieved using ImageScope software and analyzed as a TIFF image using public domain image software: ImageJ: Image Processing and Analysis in Java available from http://rsb.info.nih.gov/ij/. Retinas were manually traced for each eye and area was measured in pixels. For time point, PND8, TAg positive cells in the retina were manually counted under a 40× inverted microscope (Leica DMLB) and for PND28 and PDN84, the traced retinas were converted into an 8-bit format, and using a manually selected threshold tool, the tumor area (DAB stained) within the selected retina was highlighted and measured by the program in pixels. Total retina and tumor areas of all 5–8 analyzed sections per retina in one eye per animal were estimated calculating for percent tumor area [(tumor area in pixels/retina area in pixels) * 100]. For PND8, number of tumor cells per retinal area was used instead. BrdU positive cells were measured in pixels and quantified as an average/tumor area at PND84. Positively stained apoptotic cells were also analyzed at PND84 and manually counted per section obtaining an average number per section.

### Statistical analysis

Five animals per genotype were analyzed at PND8 and PND28. Seven animals of *Cdh11^+/+^*;*TAg^+/-^* genotype, eight animals of *Cdh11^+/-^*;*TAg^+/-^* genotype, and ten animals of *Cdh11^-/-^*;*TAg^+/-^* genotype, were analyzed at PND84. The Kruskal-Wallis (K-W) Test was the main statistical method used to investigate differences in tumor and retinal size between the genotypes at various ages. Statistical analyses were performed using SAS version 9.1 (SAS Institute, Cary, NC). All tests are two-sided and p-values equal or less than 0.05 were considered statistically significant.

### Cell lines and siRNA knockdown experiments

*Cdh11* was knocked down in the TAg-RB derived cell line T+539 using three different stealth siRNAs: MSS202865 (siRNA #1), MSS202866 (siRNA #2) and MSS202867 (siRNA #3) (Invitrogen Cat# 1320003), using GL-2 vector siRNA (Qiagen) as a control. T+539 cells were transfected in triplicate with the siRNA at time of plating, using media without the addition of penicillin and streptomycin. The procedure included transfection of 125 pmol of each siRNA oligo in Lipofectamine 2000 (Invitrogen), in a total of 2 ml plating medium. Cells were incubated for 24 hrs, 48 hrs, 72 hrs, 5 days, 7 days or 10 days. Knockdown was confirmed by immunoblot or RT-PCR for *Cdh11* (see below). Ideal inhibition was achieved 7 and 10 days post-transfection.

### RNA isolation and RT–PCR

RNA was isolated from the T+539 cell lines using the Trizol method. RNA was isolated from paraffin embedded tissue using modified GTC (guanidine isothyocionate)/ proteinase K protocol. In short tissue was deparafinized through series of incubation in xylene and 100% ethanol followed by incubation in 1M GTC/6 mg/ml proteinase K solution for 6 hrs. GTC/proteinase K was removed by phenol extraction and RNA was precipitated by equal volume of isopropanol.

Primers used for RT-PCR analysis were as follows: m*Cdh11*: forward: 5′ atg agc ctc cca tgt tct tg 3′, and reverse: 5′ggg tga tcg ctc tca cag at 3′; mKi67: forward: 5′ agc ctg tga ggc tga gac at 3′, and reverse: 5′ ttt ctg cca gtg tgc tgt tc 3′; mPCNA: forward: 5′gaa ggc ttc gac aca tac cg 3′ , and reverse: 5′ cag cat ctc caa tgt ggc ta 3′; mTBP: forward: 5′ agc aac tgc agc agc ctc agt aca 3′, and reverse: 5′ tct tcc tga atc cct tta aga tg 3′; mb-catenin: forward: 5′ caa gat gat ggt gtg cca ag 3′, and reverse: 5′ ctg cac aaa caa tgg aat gg 3′.

### Protein isolation and immunoblot

Protein isolation and immunoblot analysis were performed as described previously [13]. Dilutions for cadherin-11, caspase-3 and β-catenin antibodies used in immunoblot analysis are included in Table 1.

## Supporting Information

Figure S1 No gross differences were revealed in differentiation of retinal cell types, proliferation or expression of cadherin-2 between retinas of *Cdh11* ^+/+^ *Cdh11^+^* ^/-^ and *Cdh11* ^-/-^ littermate mice. All INL cell types were assayed to detect disruptions in retinal phenotype of *Cdh11^+/+^* versus *Cdh11* ^-/-^ littermates. Retinal cell type markers for bipolar & progenitor (Chx-10), horizontal (160 kDa), amacrine (HPC-1) and Müller glia (CRALBP) showed no evident change at developmental time points (A) ED18.5, (B) PND3 and (C) PND6. As well, no gross changes were seen in proportion of S-phase cells (via BrdU incorporation) or cadherin-2 expression.
(3.65 MB TIF)

Figure S2 β-catenin protein and mRNA levels increase after knockdown of *Cdh11*. (A) Knockdown of *Cdh11* by 2 out of 3 stealth siRNAs targeted to *Cdh11* increased expression levels of β-catenin analyzed via immunoblot in the cadherin-11 positive TAg-RB cell line, T+539. (B) Following *Cdh11* knockdown with siRNA #3, mRNA analysis showed an increase in β-catenin expression levels in the TAg-RB cell line T+539. (C) RT-PCR for β-catenin was performed on RNA isolated from TAg-RB tumours from paraffin-embedded retinal sections of PND84 *Cdh11^+/+^Tag^+/-^* and *Cdh11^-/-^ TAg^+/-^* mice. β-catenin was upregulated in the *Cdh11^-/-^ TAg^+/-^* mice relative to the *Cdh11^+/+^Tag^+/-^* mice.
(0.50 MB TIF)

Figure S3 Proliferation markers Ki67 and PCNA are not affected by knockdown of *Cdh11.* RT-PCR for *Cdh11*, Ki67, PCNA and TBP was performed on RNA isolated from the TAg-RB cell line T+539 treated with scrambled or *Cdh11* siRNA #3. *Cdh11* knockdown had no observable effect on expression of proliferation markers Ki67 and PCNA.
(0.20 MB TIF)
