# Supplementary figures and images for: Cdh11 Acts as a Tumor Suppressor in a Murine Retinoblastoma Model by Facilitating Tumor Cell Death
Source: PLoS Genet. 2010 Apr 22;6(4):e1000923. doi: 10.1371/journal.pgen.1000923 (PMC2858707; doi:10.1371/journal.pgen.1000923)

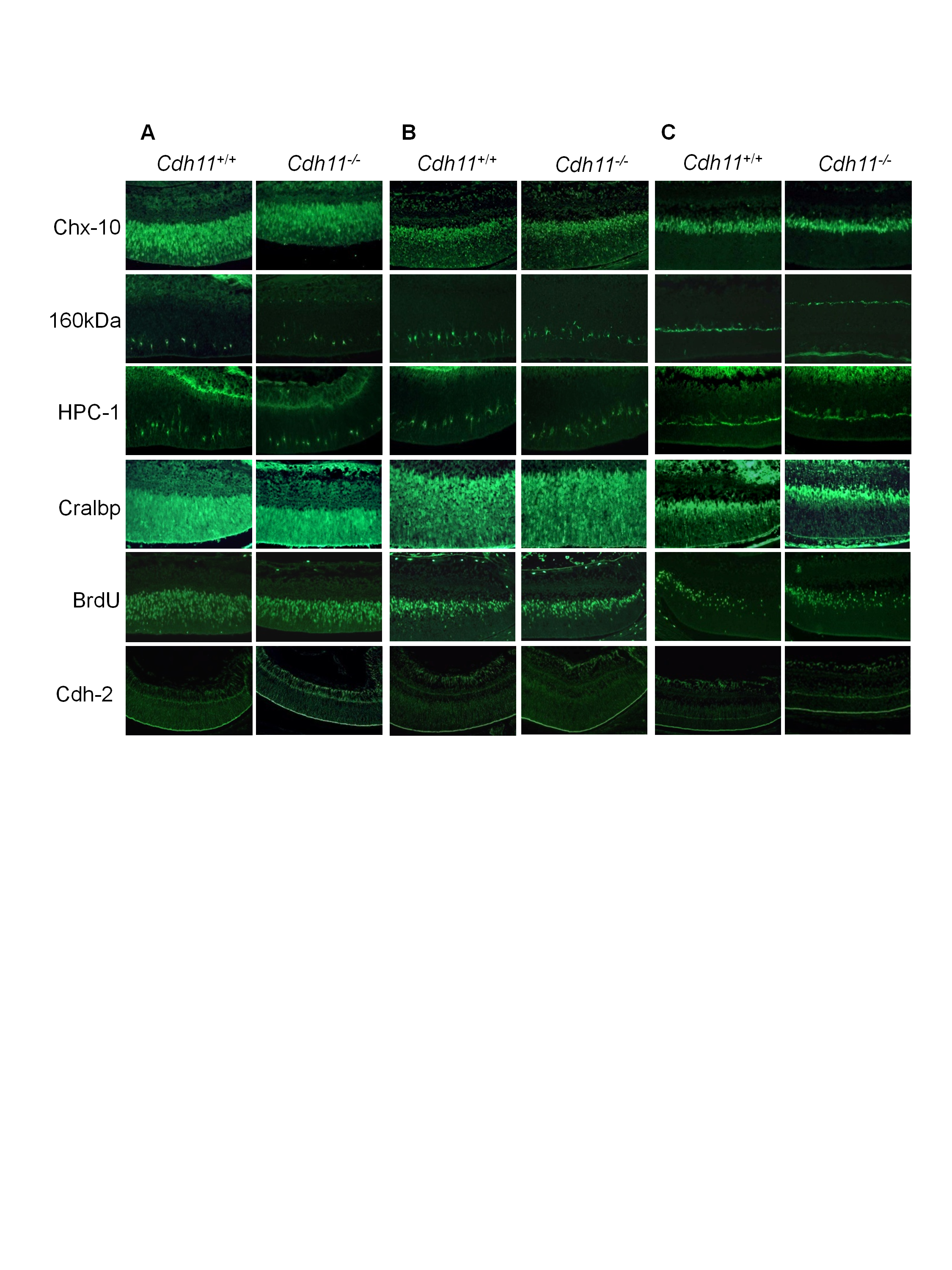

Supplement: Figure S1 — No gross differences were revealed in differentiation of retinal cell types, proliferation or expression of cadherin-2 between retinas of Cdh11 +/+ Cdh11+ /- and Cdh11 -/- littermate mice. All INL cell types were assayed to detect disruptions in retinal phenotype of Cdh11+/+ versus Cdh11 -/- littermates. Retinal cell type markers for bipolar & progenitor (Chx-10), horizontal (160 kDa), amacrine (HPC-1) and Müller glia (CRALBP) showed no evident change at developmental time points (A) ED18.5, (B) PND3 and (C) PND6. As well, no gross changes were seen in proportion of S-phase cells (via BrdU incorporation) or cadherin-2 expression. (3.65 MB TIF) [file pgen.1000923.s001.tif]

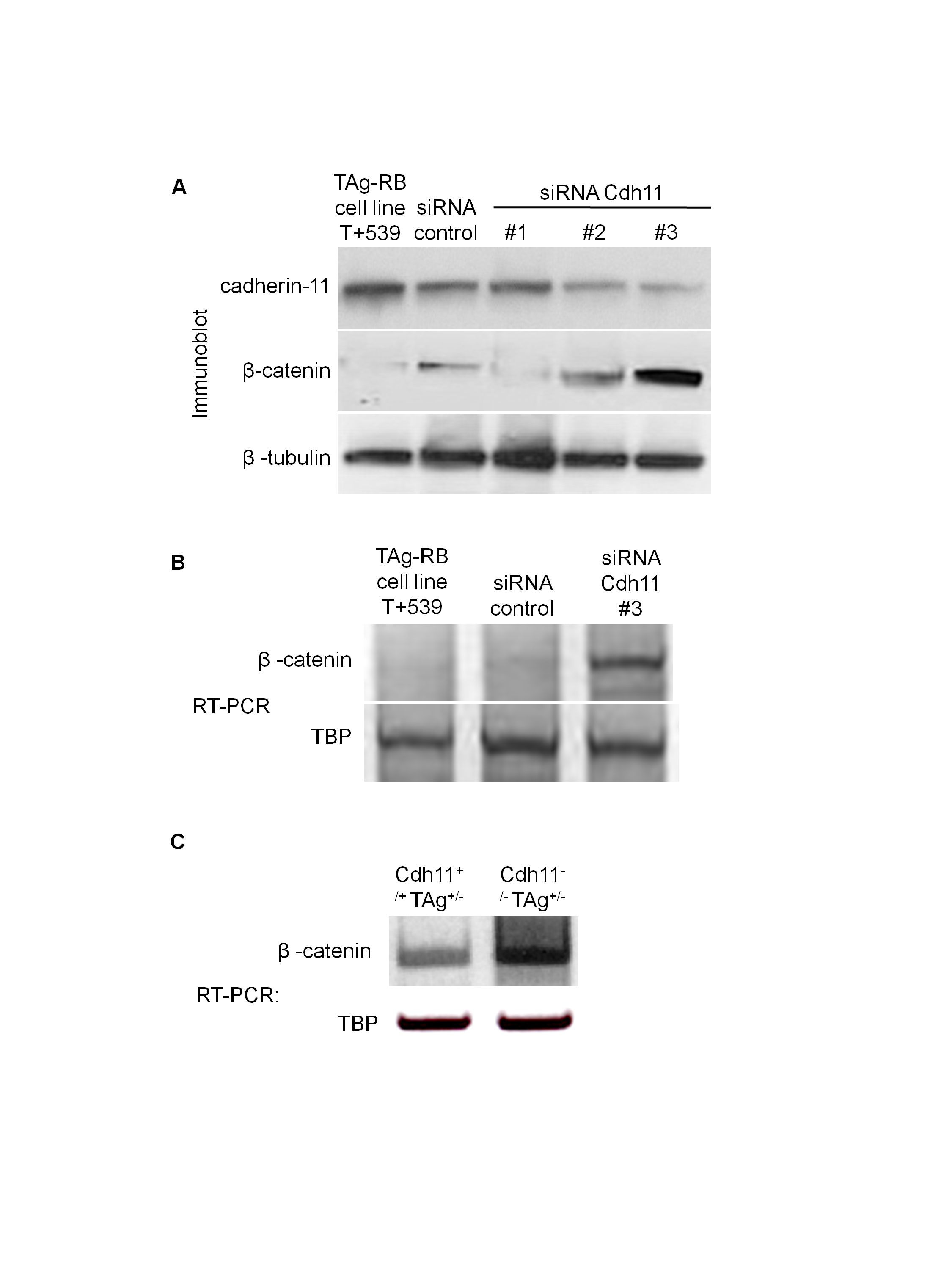

Supplement: Figure S2 — β-catenin protein and mRNA levels increase after knockdown of Cdh11. (A) Knockdown of Cdh11 by 2 out of 3 stealth siRNAs targeted to Cdh11 increased expression levels of β-catenin analyzed via immunoblot in the cadherin-11 positive TAg-RB cell line, T+539. (B) Following Cdh11 knockdown with siRNA #3, mRNA analysis showed an increase in β-catenin expression levels in the TAg-RB cell line T+539. (C) RT-PCR for β-catenin was performed on RNA isolated from TAg-RB tumours from paraffin-embedded retinal sections of PND84 Cdh11+/+Tag+/- and Cdh11-/- TAg+/- mice. β-catenin was upregulated in the Cdh11-/- TAg+/- mice relative to the Cdh11+/+Tag+/- mice. (0.50 MB TIF) [file pgen.1000923.s002.tif]

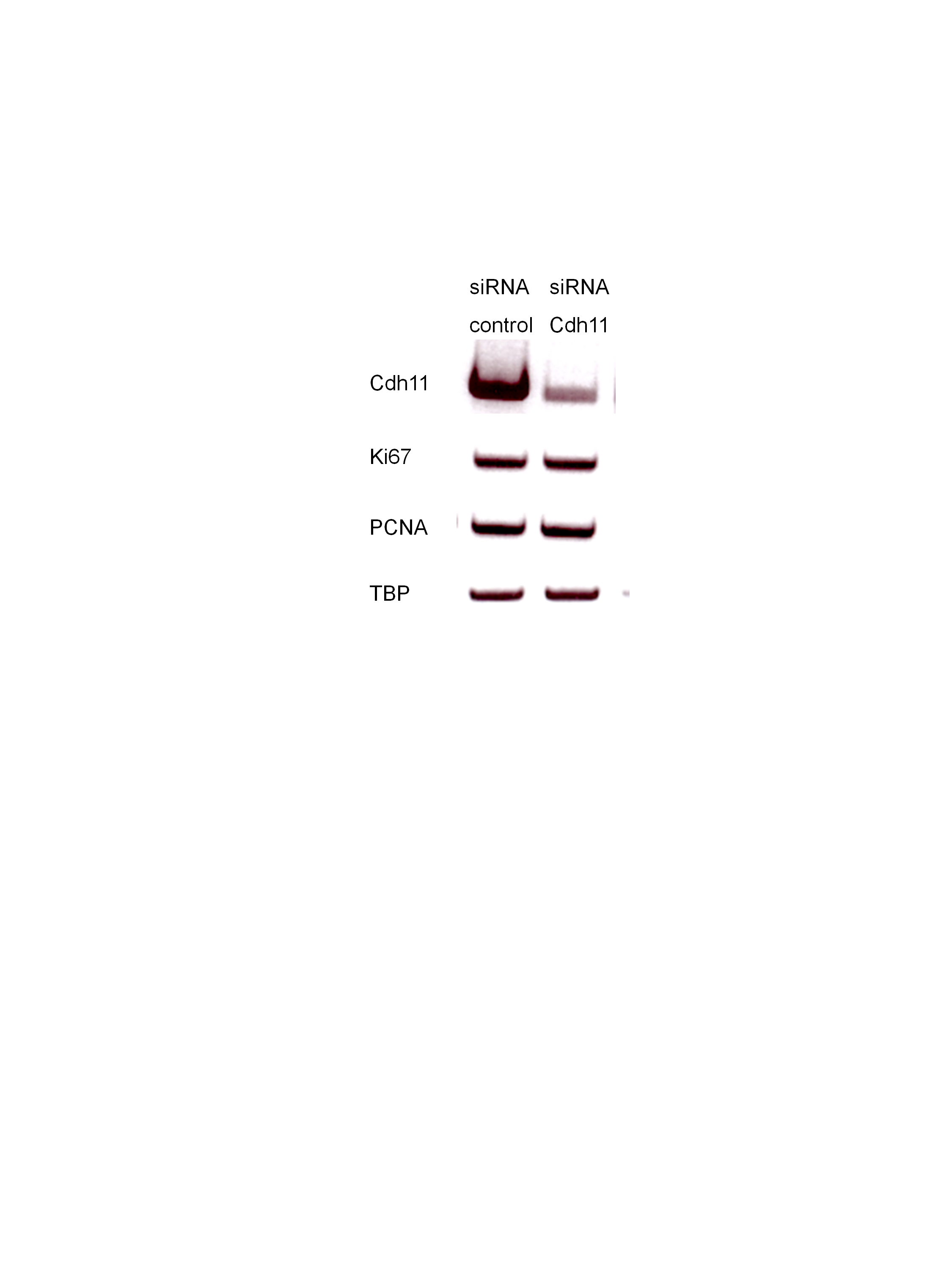

Supplement: Figure S3 — Proliferation markers Ki67 and PCNA are not affected by knockdown of Cdh11. RT-PCR for Cdh11, Ki67, PCNA and TBP was performed on RNA isolated from the TAg-RB cell line T+539 treated with scrambled or Cdh11 siRNA #3. Cdh11 knockdown had no observable effect on expression of proliferation markers Ki67 and PCNA. (0.20 MB TIF) [file pgen.1000923.s003.tif]
